# Comment on “The influence of familiarity between the surgeon and their assistant on patient outcomes: a prospective observational cohort study”

**DOI:** 10.1097/JS9.0000000000002849

**Published:** 2025-06-24

**Authors:** Xiaoping Sheng, Hongsheng Zhan, Zhengyan Li, Guoqing Du

**Affiliations:** a Shanghai Municipal Hospital of Traditional Chinese Medicine, Shanghai University of Traditional Chinese Medicine, Shanghai, China; b Shi’s Center of Orthopedics and Traumatology, Shuguang Hospital Affiliated to Shanghai University of Traditional Chinese Medicine, Shanghai, China

*Dear Editor*,

With interest, we read the article by Stelzl *et al*^[1]^ demonstrating a significant inverse association between surgeon-assistant dyad familiarity and surgical adverse events. The study’s rigorous methodology—including multivariable adjustments, sensitivity analyses, and case-mix variable control—revealed a logarithmic “teaming curve” wherein complications markedly decreased within the first 15 collaborations. This multispecialty analysis of 8,546 operations across 14 departments provides actionable evidence for optimizing operating room (OR) team stability while addressing a critical gap in surgical teamwork literature. We commend the authors for this valuable contribution. However, several methodological considerations warrant discussion.

Firstly, while the findings are compelling, the observational design inherently precludes causal inferences. Unmeasured confounders—particularly potential case selection bias such as preferential assignment of familiar teams to lower-risk cases—cannot be excluded. Secondly, the comparability of composite postoperative endpoints remains problematic. Surgical adverse events exhibit inherent heterogeneity due to variations in patient comorbidities, procedural complexity, and institutional protocols. Although composite endpoints provide holistic efficiency, aggregating complications ranging from surgical-site infections to organ failure may obscure procedure-specific or severity-driven effects. Stratifying outcomes by complication type or severity would be highly appropriate to clarify whether familiarity disproportionately benefits certain events. Thirdly, the operationalization of familiarity merits scrutiny. Quantifying teamwork synergy solely through prior collaboration counts may overlook qualitative dimensions such as interpersonal rapport or shared mental models developed through simulation training. Additionally, excluding dyads with >50 collaborations precludes understanding potential performance plateaus at higher familiarity levels. Fourthly, the exclusive focus on surgeon-assistant dyads neglects the broader OR ecosystem. Familiarity among anesthesiologists, nurses, and technicians may have synergistic effects on outcomes-a dimension requiring integrated analysis^[2]^. Finally, generalizability is constrained by the French healthcare context (e.g., standardized resident rotations) and exclusion of supervising roles for junior surgeons, limiting applicability to systems with divergent training paradigms.

Future research could productively explore causal mechanisms through randomized trials comparing familiar versus unfamiliar dyads, ideally integrated with qualitative assessments of communication and workflow dynamics. It would also be valuable to extend investigations to include anesthesiologists and nurses, thereby enabling a more holistic evaluation of operating room team familiarity. Given practical constraints, intervention studies might examine whether simulation training or structured briefings could potentially accelerate teamwork synergy in ad hoc teams. Then, economic analyses would be warranted to systematically weigh the benefits of improved patient outcomes against the logistical implications of maintaining stable surgical teams. Lastly, with the increasing integration of artificial intelligence (AI) in surgical environments, future studies should investigate how AI tools—such as real-time decision support systems, predictive analytics, or automated workflow monitoring—might enhance team coordination, optimize task allocation, and support situational awareness, especially in teams with low baseline familiarity. The recently published TITAN guideline provides a framework to ensure reproducibility and ethical standards in AI-supported research and will be valuable for future studies, especially in shaping scientific workflows^[3]^.

In conclusion, this study crucially underscores OR team stability as a modifiable factor in patient safety. Hospitals could consider prioritize strategies to maintain consistent surgeon-assistant dyads, particularly for early-career assistants.

## Data Availability

Not applicable.
